# Prolonged Infectivity of SARS-CoV-2 in Fomites

**DOI:** 10.3201/eid2609.201788

**Published:** 2020-09

**Authors:** Boris Pastorino, Franck Touret, Magali Gilles, Xavier de Lamballerie, Rémi N. Charrel

**Affiliations:** Unité des Virus Émergents, Marseille, France.

**Keywords:** COVID-19, severe acute respiratory syndrome coronavirus 2, SARS-CoV-2, SARS, 2019 novel coronavirus disease, coronavirus disease, zoonoses, viruses, coronavirus, fomites

## Abstract

We spotted severe acute respiratory syndrome coronavirus 2 on polystyrene plastic, aluminum, and glass for 96 hours with and without bovine serum albumin (3 g/L). We observed a steady infectivity (<1 log_10_ drop) on plastic, a 3.5 log_10_ decrease on glass, and a 6 log_10_ drop on aluminum. The presence of proteins noticeably prolonged infectivity.

Severe acute respiratory syndrome coronavirus 2 (SARS-CoV-2) has spread worldwide, demonstrating a great potential for direct and indirect transmission between humans. Coronaviruses can keep their infectivity in fomites and thus can remain infectious on dry surfaces for hours (*1*,*2*). However, limited data are available for SARS-CoV-2 (*1*). Specifically, there are no data about the role of interfering substances such as proteins on SARS-CoV-2 infectivity in the environment. We evaluated the stability and infectivity of SARS-CoV-2 deposited on polystyrene plastic, aluminum, and glass for 96 hours at 45%–55% relative humidity (recommended for indoor living spaces by the American Society of Heating, Refrigeration and Air Conditioning Engineers) and 19°C –21°C temperature range using a 10^6^ 50% tissue culture infectivity dose (TCID_50_)/mL inoculum.

We inoculated SARS-CoV-2 at a multiplicity of infection of 0.001 onto Vero E6 cells incubated at 37°C in 5% CO_2_ for 72 h (Appendix). We collected the supernatant and clarified it by spinning at 1500 × *g* for 10 min. We prepared aliquots and stored them at −80°C before titration. We measured virus infectivity using TCID_50_. We diluted the inoculum in cell culture medium containing 5% fetal bovine serum (FBS; final protein concentration 1.8 g/L) to 10^6^ TCID_50_/mL. For experiments with a higher protein concentration, we used a concentrated bovine serum albumin (BSA) solution (40 g/L) to result in a final protein concentration of 11.4 g/L. We measured virus infectivity sequentially on polypropylene plastic, aluminum, and glass slides. We deposited a 50-μL drop in triplicate on the various surfaces (≈1 cm^2^ per piece) and recovered them sequentially to quantify viable infectious virions by endpoint titration on Vero E6 cells. The limit of detection for the assays was 10^0.5^ TCID_50_/mL.

We conducted our experiments with and without BSA to mimic the protein content within body fluids of the respiratory system such as cough droplets, sputum, and airway mucosal secretions (*3*). Final protein concentration was 1.8 g/L without BSA conditions and 11.4 g/L with BSA conditions. We observed 3 different profiles, depending on surface type: a 3.5 log_10_ decrease over 44 h on glass (Figure, panel A), a steady infectivity with a <1 log_10_ drop over 92 h on polystyrene plastic (Figure, panel B), and a sharp 6 log_10_ drop in <4 h on aluminum (Figure panel C). The probable adsorption of viral particles onto a plastic polystyrene surface was associated with prolonged infectivity, whereas a high drop on aluminum was observed as in previously published data on SARS-CoV, adenovirus, or poliovirus (*4*,*5*). Our results have also shown higher stability for SARS-CoV-2 on polystyrene plastic, with or without BSA, in comparison with a recent study (*1*); this variation could be explained by a different type of plastic used in the 2 studies. Regardless of the type of surface, virus infectivity decreased ≈1 log_10_ within 2 h (Table). To study SARS-CoV-2 stability in solution, we titrated cell culture supernatants containing 10^6^ TCID_50_/mL every 24 h for 96 h. We found that SARS-CoV-2 was very stable, showing an overall decreased infectivity <1.4 log_10_ reduction, results similar to those described for SARS-CoV (Appendix Figure) (*4*).

**Figure:**
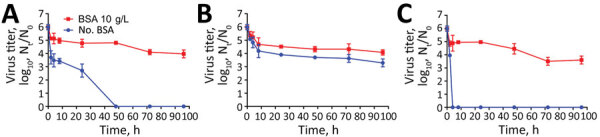
Viability of severe acute respiratory syndrome coronavirus 2 on various surfaces and in suspension. Viruses were applied to glass (A), polystyrene plastic (B), and aluminum (C) at 45%–55% relative humidity at 19°C–21°C for 96 h. The titer of viable virus is expressed as TCID_50_/mL of collection medium. All samples were quantified by endpoint titration on Vero E6 cells with a limit of detection of ≈10^0.5^ TCID_50_/mL. TCID_50_, 50% tissue culture infectivity dose.

**Table:** SARS-CoV-2 titer values for different materials*

Time, h	Material	SARS-CoV-2 in suspension

Glass			Aluminum			Plastic	
No BSA	BSA 10 g/L	No BSA	BSA 10 g/L	No BSA	BSA 10 g/L
0	6 ± 0.2								
2	3.7 ± 0.5	5.1 ± 0.1		4 ± 0.1	4.8 ± 0.2		5.1 ± 0.1	5.4 ± 0.3	
4	3.5 ± 0.5	5.1 ± 0.4		ND	4.8 ± 0.5		4.8 ± 0.4	5.2 ± 0.4	
8	3.4 ± 0.2	4.9 ± 0.2		ND	4.9 ± 0.1		4.2 ± 0.5	4.6 ± 0.5	
24	2.7 ± 0.5	4.7 ± 0.3		ND	4.9 ± 0.1		3.8 ± 0.1	4.5 ± 0.1	5.99
48	ND	4.8 ± 0.1		ND	4.4 ± 0.4		3.7 ± 0.1	4.3 ± 0.2	4.99
72	ND	4.1 ± 0.2		ND	3.4 ± 0.3		3.6 ± 0.3	4.3 ± 0.4	3.99
96	ND	3.9 ± 0.3		ND	3.6 ± 0.3		3.3 ± 0.3	4.1 ± 0.2	3.99
Half-life	17	>96		2.5	>96		>96	>96	>96

*Values are mean value of 3 replicates ± SD. BSA, bovine serum albumin; ND, not detectable; SARS-CoV-2, severe acute respiratory syndrome coronavirus 2.

Our data showed that SARS-CoV-2 infectivity was remarkably preserved in the presence of proteins, regardless of the type of surface. A final concentration of 11.4 g/L of proteins, as used in our study, closely mimics that of respiratory fluids, which possess protein concentrations of a similar order of magnitude. However, the respiratory body fluids are complex media including not only proteins, but also enzymes and mucins (present in mucus) that may have a negative effect on virus infectivity. Regarding viral load measurement, the reason for avoiding the use of molecular techniques such as reverse transcription PCR is that despite that they allow quantification of RNA copies and determination of RNA decay, they cannot measure residual infectivity on various surfaces.

The protective effect of proteins had already been described for pandemic SARS-CoV or suggested for influenza A(H1N1) virus, but with less notable effects (*4*,*6*). As illustrated in other virus models (*7*), interfering substances such as proteins influenced the resistance of SARS-CoV-2 to drying and thus its persistence in the environment.

In conclusion, we showed that a moderate protein concentration in droplets markedly increased the infectivity of SARS-CoV-2, suggesting that a protein-rich medium like airway secretions could protect the virus when it is expelled and may enhance its persistence and transmission by contaminated fomites. Accordingly, it is plausible that fomites infected with SARS-CoV-2 play a key role in the indirect transmission of coronavirus disease (COVID-19). This finding supports surface cleaning as a necessary action that should be enforced and repeated becuase it may play a key role in halting SARS-CoV-2 transmission and mitigating the COVID-19 pandemic.

Appendix Additional information on study of viability of SARS-CoV-2 in fomites.
