# Supplementary material for: Prolonged Infectivity of SARS-CoV-2 in Fomites
Source: Emerg Infect Dis. 2020 Sep;26(9):2256–7. doi: 10.3201/eid2609.201788 (PMC7454106; doi:10.3201/eid2609.201788)
Supplement: Appendix — Additional information on study of viability of SARS-CoV-2 in fomites. [file 20-1788-Techapp-s1.pdf]

Article DOI: https://doi.org/10.3201/eid2609.201788

Prolonged Viability of SARS-CoV-2 in Fomites

Appendix

Methods

Cell Line

Vero E6 cells (ATCC#CRL-1586) were grown at 37°C in 5% CO_2 with 1% penicillin/streptomycin (PS; 5000 U/mL and 5000 µg/mL; Life Technologies, https://www.thermofisher.com/us/en/home/brands/life-technologies.html) and supplemented with 1% nonessential amino acids (Life Technologies) in minimal essential medium (MEM; Life Technologies) with 5% FBS.

Viruses

The human 2019 SARS-CoV-2 strain (Ref-SKU: 026V-03883) was isolated at Charite University (Berlin, Germany) and obtained from the European Virus Archive catalog (EVA-GLOBAL H2020 project) (https://www.european-virus-archive.com). Experiments were performed in Biosafety Level 3 (BSL3) facilities.

SARS-CoV-2 Titration

SARS-CoV-2 was first propagated and titrated on Vero E6 cells. Virus stock was diluted to infect Vero E6 cells at a multiplicity of infection (MOI) of 0.001, then cells were incubated at 37°C for 24–48 h, after which medium was changed and incubation was continued for 24 h; then supernatant was collected, clarified by spinning at 1500 × *g* for 10 min, supplemented with 25 mM HEPES (Sigma, https://www.sigmaaldrich.com), and aliquoted. Aliquots were stored at –80°C before titration. Virus infectivity was measured using 50% tissue culture infectivity dose ($TCID_{50}$); briefly, when cells were at 90% confluence, 6 replicates were infected with 150 µL of tenfold serial dilutions of the virus sample and incubated for 4 days at 37°C under 5% CO_2. Cytopathic effect was read using an inverted microscope, and infectivity was expressed as $TCID_{50}$/ml based on the Karber formula (*1*).

Virus Stability on Surfaces

Surface stability was evaluted on plastic (polypropylene; Corning Inc., https://www.corning.com), aluminium (Thermo Scientific, https://www.thermofisher.com), and glass (Thermo Scientific). All experiments were conducted in a BSL-3 lab under biosafety cabinet at 45–55% relative humidity and 19–21°C. An inoculum of 10^6 $TCID_{50}$/mL was resuspended in cell culture medium (complete MEM plus 5% FBS) with or without BSA 10 g/L and placed on test surfaces. In short, 50 μL of virus was deposited on the surface ($\approx$1 cm^2 per piece) and recovered at predefined timepoints by adding 150 μL of complete medium. Three replicate experiments were performed for each surface and viable virus was immediately quantified by endpoint titration on Vero E6 cells, as described previously (*2*). The limit of detection for the assays was about $10^{0.5}$ $TCID_{50}$/mL.

Results

In suspension, SARS-CoV-2 was stable for the entire duration of the experiment (96 h) (virus titer decrease $<1.4 \log_{10}$) as already described for SARS coronavirus (Appendix Figure) (*3*).

References

1. Reed LJ, Muench H. A simple method of estimating fifty per cent endpoints. Am J Epidemiol. 1938;27:493–7. http://dx.doi.org/10.1093/oxfordjournals.aje.a118408

<jrn>2. Pastorino B, Touret F, Gilles M, Luciani L, de Lamballerie X, Charrel RN. Evaluation of chemical protocols for inactivating SARS-CoV-2 infectious samples. Viruses. 2020; 12: E624. http://dx.doi.org/10.3390/v12060624

3. Rabenau HF, Cinatl J, Morgenstern B, Bauer G, Preiser W, Doerr HW. Stability and inactivation of SARS coronavirus. Med Microbiol Immunol (Berl). 2005;194:1–6. PubMed http://dx.doi.org/10.1007/s00430-004-0219-0

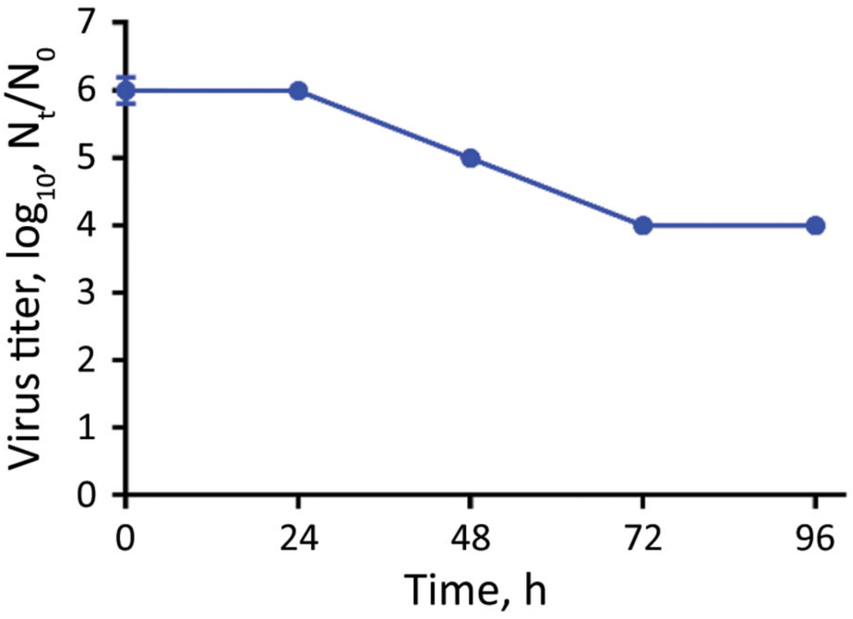

Appendix Figure. In vitro stability of SARS-CoV-2. Infected cell supernatant (complete MEM medium, 5% FCS) was incubated at room temperature in suspension and titrated every 24 hours. Values are means for 2 independent experiments.
